# Case Report: *Mycobacterium haemophilum* infection following minor trauma: diagnostic challenges and treatment implications

**DOI:** 10.3389/fmed.2026.1813633

**Published:** 2026-04-30

**Authors:** Jue Wang, Xinlong Chen, Lizhu Chen

**Affiliations:** 1 Department of Dermatology, Hospital of Chengdu University of Traditional Chinese Medicine, Chengdu, Sichuan, China; 2 Chengdu University of Traditional Chinese Medicine, Chengdu, Sichuan, China

**Keywords:** *Mycobacterium haemophilum*, non-tuberculous mycobacteria, skin and soft tissue infection, trauma, wound healing

## Abstract

**Background:**

Non-tuberculous mycobacteria (NTM) infections are increasingly recognized in clinical practice, yet diagnosis remains challenging.

**Case presentation:**

A 52-years-old immunocompetent male developed chronic finger infection after shrimp shell injury.

**Methods:**

MetaCap sequencing was used.

**Results:**

*Mycobacterium haemophilum* was identified; patient recovered after combination therapy.

**Conclusion:**

MetaCap enables early diagnosis and targeted treatment.

## Introduction

Non-tuberculous mycobacteria (NTM) infections are increasingly recognized due to rising incidence and diagnostic complexity. *Mycobacterium haemophilum* is a slow-growing pathogen causing cutaneous infections, particularly in immunocompromised individuals or after trauma (1). Its environmental distribution and intrinsic drug resistance make diagnosis and treatment challenging (2). The epidemiology of *M. haemophilum* has shifted, with increasing reports associated with procedures and chronic wounds (3). These infections often mimic common bacterial infections, leading to misdiagnosis (4, 5). Here, we report a rare case following minor trauma, highlighting the diagnostic value of MetaCap and the importance of early targeted therapy. In the context of the present case, the patient experienced prolonged symptoms characterized by persistent swelling and pain in the right index finger following a self-inflicted injury from a shrimp shell. The diagnosis of *M. haemophilum* was confirmed. This case is particularly significant as it represents one of the few documented instances of *M. haemophilum* infection following a minor trauma, providing valuable insights into the pathogen’s clinical behavior and the potential for successful treatment through targeted antibiotic therapy. The rarity of such cases, coupled with the distinct treatment response observed here, highlights the critical need for clinical vigilance against NTM infections. MetaCAP has the potential to significantly improve their early diagnosis and thereby guide timely therapeutic intervention.

## Case presentation

**FIGURE 1:**
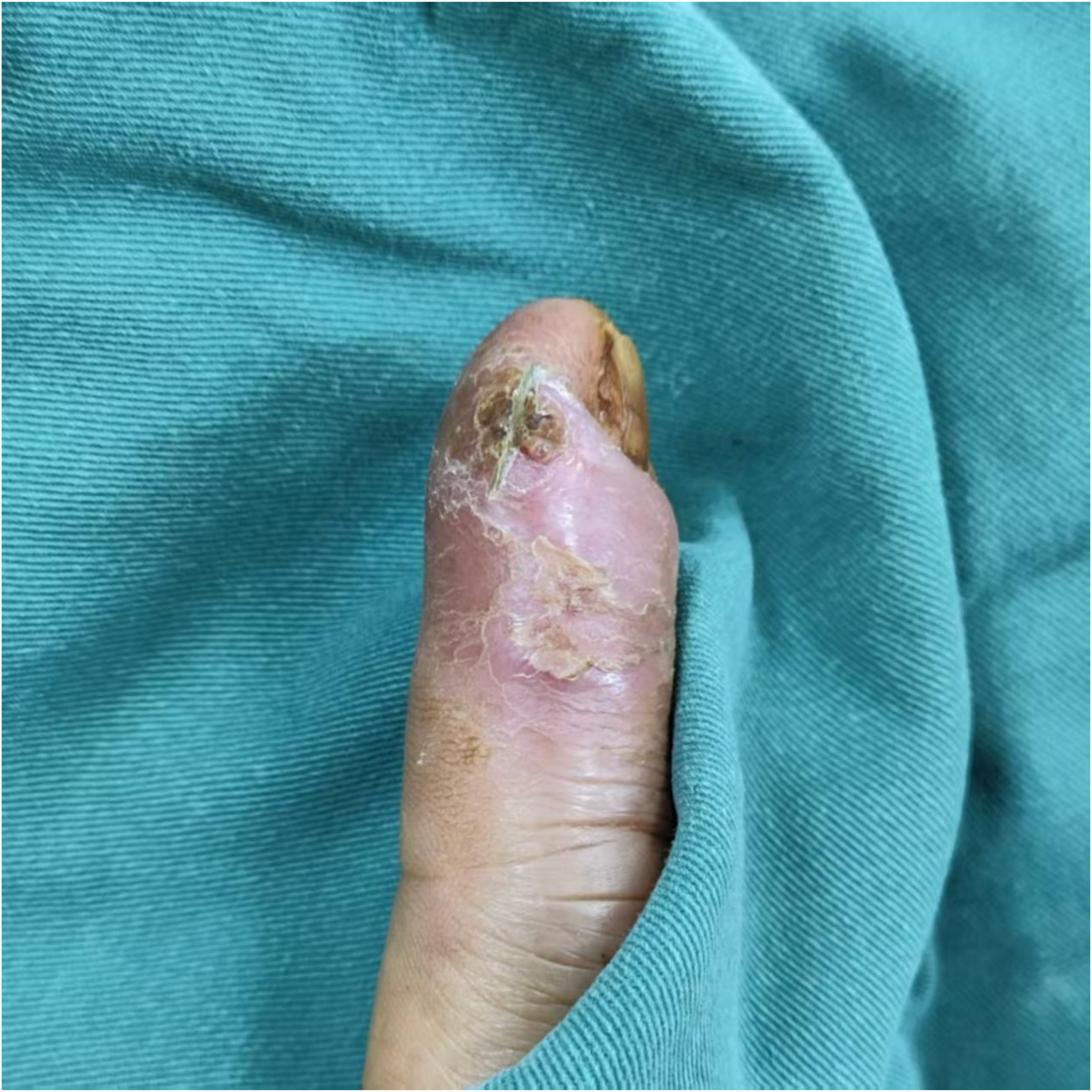
The wound upon admission. The right index finger presented with an area of erythema, swelling, and a yellowish crust. Underlying the crust, viscous, yellowish purulent fluid was observed.

The patient was a 52-years-old male with no history of diabetes, malignancy, HIV infection, or immunosuppressive therapy. He denied long-term corticosteroid use. He worked as an office worker living in southern China and had no known occupational aquatic exposure. He sustained a puncture injury to the right index finger from a shrimp shell. Initial treatment with levofloxacin resulted in partial improvement, followed by recurrence. Approximately 50 days after the injury, he presented with persistent local inflammation and delayed wound healing. Prior to referral, no targeted antimicrobial therapy against non-tuberculous mycobacteria had been administered. On physical examination, the lesion was located over the proximal interphalangeal joint of the right index finger. It measured approximately 2.0 cm × 1.5 cm and presented as an erythematous, swollen plaque with an overlying yellowish crust. Upon removal of the crust, a small amount of viscous purulent exudate was observed. The surrounding skin was warm and tender. No discrete nodules, fluctuant abscess, deep ulceration, lymphatic spread, or sporotrichoid pattern was noted (Figure 1).

**FIGURE 2:**
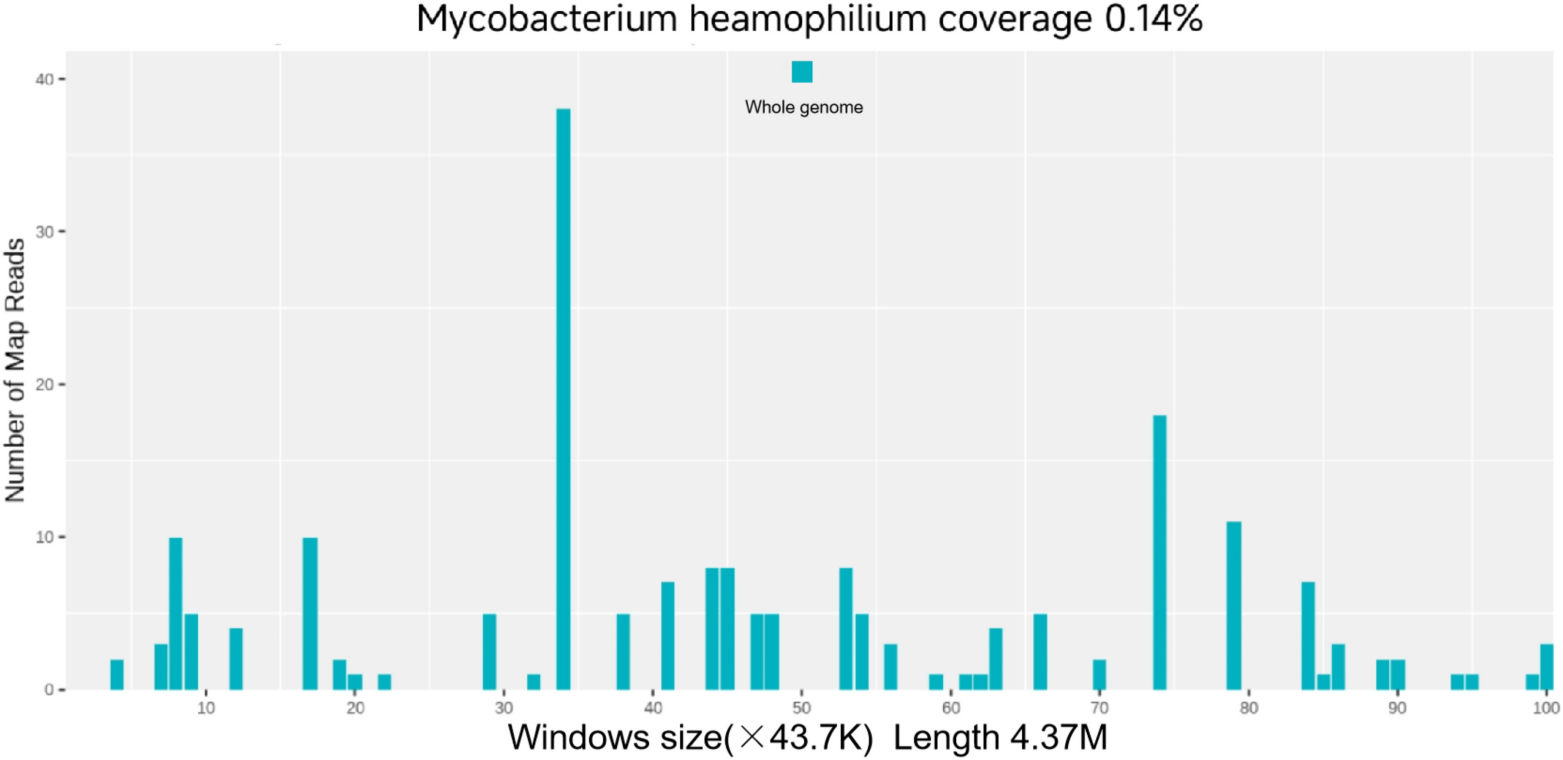
Distribution map of *M. haemophilum* detected by Metacap in skin lesion tissue. MetaCAP detected *M. haemophilum* with 188 RPM reads (Reads per Million sequence reads) and a 99% confidence level.

Relevant laboratory tests on admission, including complete blood count, liver and renal function, coagulation profile, hepatitis B, hepatitis C, syphilis, HIV, glycated hemoglobin, procalcitonin, urinalysis, and stool examination, showed no significant abnormalities (Figure 2). Culture of pus and tissue samples yielded a small amount of coagulase-negative Staphylococcus, considered non-specific, while acid-fast staining was negative. Histopathology of the right index finger lesion demonstrated granulation tissue, collagen proliferation, and inflammatory cell infiltration, suggesting an infectious process. Because conventional findings were inconclusive and antimicrobial susceptibility testing could not be performed due to failure to isolate the causative organism, MetaCap was performed on lesional tissue for microbial nucleic acid analysis, which identified *M. haemophilum* as the causative pathogen.

Differential diagnoses included other non-tuberculous mycobacterial infections, particularly *Mycobacterium marinum*, as well as sporotrichosis, nocardiosis, and rapidly growing mycobacterial infection. These were considered less likely based on the lesion morphology, absence of a sporotrichoid pattern, and the final sequencing result.

**FIGURE 3:**
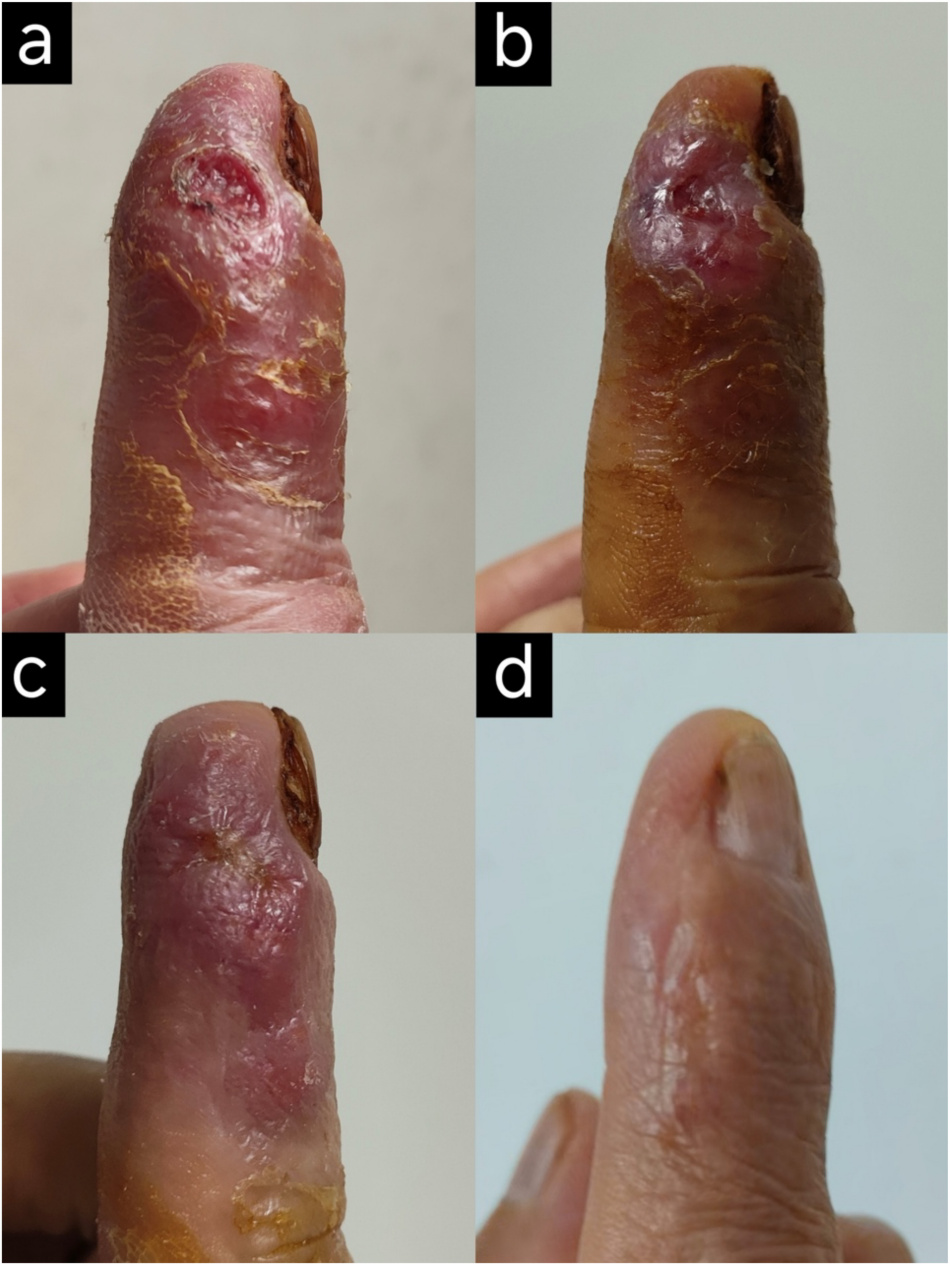
The healing process. **(a)** After 7 days of targeted treatment, debridement and dressing change of the right index finger still revealed the presence of necrotic tissue and purulent discharge. **(b)** As treatment continued, a significant reduction in wound size and exudate was observed by day 14. **(c)** The wound was completely closed by day 21, although the finger remained erythematous, swollen, and warm to the touch. **(d)** At the 5-months post-discharge follow-up, the wound on the right index finger was well-healed.

The patient received a triple antibiotic regimen consisting of clarithromycin (0.5 g, po, BID), moxifloxacin (0.4 g, ivgtt, QD), and rifampicin (0.45 g, po, QD) based on known susceptibility patterns of *M. haemophilum*. Previous studies have shown high *in vitro* susceptibility of this organism to macrolides, rifamycins, and fluoroquinolones, with clarithromycin showing consistent activity and high susceptibility rates reported for ciprofloxacin and rifampicin (6). Antimicrobial susceptibility testing was not performed because the causative organism was not successfully cultured under available laboratory conditions. During treatment, the wound evolved from necrotic tissue and exudation (Figures 3a,b) to scab formation with reduced erythema and swelling (Figure 3c). After 21 days, the regimen was changed to oral clarithromycin (0.5 g, po, BID), ciprofloxacin (0.5 g, po, TID), and rifampicin (0.45 g, po, QD) because of clinical improvement and hospital discharge. After 3 months, due to abnormal liver function tests, the regimen was adjusted to clarithromycin and ciprofloxacin. After 5 months of treatment, the wound demonstrated marked healing with complete resolution of local erythema and swelling (Figure 3d). At 1-year follow-up, liver function remained normal, and no infection-related complications or recurrence were observed.

## Discussion

Chronic infections caused by atypical mycobacteria, particularly *M. haemophilum*, present a significant challenge in clinical diagnosis and management. This case highlights a unique presentation following a minor injury, diverging from the common understanding of typical infections linked to more frequently encountered pathogens. Previous literature has documented instances of *M. haemophilum* infections, particularly in immunocompromised individuals, yet the presentation following minor trauma is less frequently reported. For instance, studies have described *M. haemophilum* infections after cosmetic procedures and minor surgical interventions, emphasizing its opportunistic nature in vulnerable populations (7, 8). Importantly, *M. haemophilum* is considered an environmental organism, with water-related environments regarded as its most likely reservoir, although its exact ecological niche remains incompletely defined. Infections have been associated with exposure to contaminated water sources or aquatic environments, suggesting that transmission may occur through direct inoculation following disruption of the skin barrier. In the present case, the shrimp-shell injury may have served as a portal of entry, potentially introducing the organism from a marine-associated environment into the subcutaneous tissue (9, 10).

Moreover, this case underscores the necessity of utilizing advanced diagnostic techniques, such as high-throughput sequencing. In this study, MetaCap sequencing was performed on purulent secretion from the lesion. MetaCap is a probe capture–based pathogen nucleic acid sequencing approach that combines host depletion with million-probe–based enrichment to reduce host background interference and improve pathogen detection. Bioinformatic analysis included quality control, host sequence removal, and alignment to a comprehensive microbial database. A total of 188 reads corresponding to *M. haemophilum* were identified, with high confidence (99%), supporting the diagnosis. This method has previously demonstrated effectiveness in identifying atypical mycobacterial infections (11, 12), particularly when conventional culture is difficult. However, limitations such as potential contamination, lack of absolute quantification, and the need for clinical correlation in interpreting detected organisms should still be considered. The resistance patterns associated with *M. haemophilum* further complicate treatment, necessitating a combination of tailored antibiotic regimens (13, 14). This case serves as a critical reminder for clinicians regarding the importance of thorough evaluation in similar scenarios, particularly when faced with chronic infections that may not respond to standard treatment protocols (15).

The diagnostic challenges associated with infections caused by atypical mycobacteria, specifically *M. haemophilum*, are highlighted by this case, which underscores the potential for misdiagnosis stemming from initial clinical presentations that may not align with typical infection profiles. The patient’s symptoms, following a seemingly minor injury, illustrate the diagnostic pitfall of underestimating the infectious potential of such injuries. This aligns with findings from previous studies which emphasize that minor trauma can lead to significant infections, particularly in immunocompromised patients, as documented in a literature review on chronic infections (16). The incorporation of advanced techniques such as high-throughput sequencing has proven invaluable in accurately identifying atypical pathogens, facilitating timely and appropriate interventions (17).

Furthermore, the resistance patterns exhibited by *M. haemophilum* underscore the complexity of treatment regimens required for effective management. The documented multi-drug resistance necessitates a tailored approach, often involving combinations of antibiotics based on susceptibility testing to achieve favorable outcomes (18, 19). The implications for long-term therapy, particularly in terms of monitoring liver function due to the hepatotoxic potential of certain antibiotics, are critical considerations for clinicians managing such infections. This case serves as a compelling reminder of the necessity for heightened awareness and education regarding atypical mycobacterial infections, particularly in healthcare settings where early intervention could significantly improve patient outcomes (20, 21) (Table 1).

**TABLE 1:** Summary of reported cutaneous *M. haemophilum* infections and comparison with the present case.

Author (year)	Immune status	Exposure history	Clinical presentation	Lymphatic spread	Diagnostic method	Treatment	Outcome
**Saubolle** and **Sussland (22)**	Immunocompromised	None reported	Nodules, ulcers	Occasionally present	Culture	Macrolide-based therapy	Improved
**Lindeboom et al. (23)**	Immunocompromised (children)	None reported	Cervicofacial/cutaneous lesions	Rare	Culture + PCR	Combination therapy	Resolved
**Weitzul et al. (24)**	Mixed	Trauma/environmental exposure	Nodules, plaques, ulcers	Variable	Culture	Combination antibiotics	Improved
**Franco-Paredes** **et al. (2)**	Mixed	Environmental exposure	Cutaneous and soft tissue lesions	Variable	Culture + molecular methods	Multidrug therapy	Variable
**Present case (2024)**	Immunocompetent	Shrimp injury (aquatic exposure)	Erythema, swelling, purulent exudate	Absent	MetaCap sequencing	Clarithromycin + rifampicin + fluoroquinolone	Resolved

PCR, polymerase chain reaction; MetaCap, metagenomic capture sequencing.

This study has several limitations. First, it represents a single-patient observation, which limits the generalizability of the findings. Second, culture confirmation under specialized conditions was not achieved, and antimicrobial susceptibility testing could not be performed because the causative organism was not successfully isolated. Finally, while metagenomic capture sequencing (MetaCap) facilitated pathogen identification in this case, it does not provide phenotypic susceptibility data and should be interpreted cautiously in conjunction with clinical and pathological findings.

## Conclusion

The case presented underscores the importance of recognizing atypical mycobacterial infections, particularly *M. haemophilum*, in clinical practice. The unique circumstances surrounding this patient’s infection highlight critical learning points relevant to both diagnosis and management. This emphasizes the necessity for clinicians to maintain a high index of suspicion when confronted with chronic wounds or persistent symptoms following minor trauma. Moreover, the successful identification of *M. haemophilum* through advanced diagnostic techniques, such as high-throughput sequencing, illustrates the need for ongoing innovation in microbiological evaluation to facilitate timely interventions.

## Data Availability

The original contributions presented in this study are included in this article/supplementary material, further inquiries can be directed to the corresponding author.
